# Supplementary material for: Rearing condition influences gene expression in postlarval American lobster (Homarus americanus)
Source: PLoS One. 2024 Jul 18;19(7):e0307169. doi: 10.1371/journal.pone.0307169 (PMC11257295; doi:10.1371/journal.pone.0307169)
Supplement: S3 File — (DOCX) [file pone.0307169.s003.docx]

**DNA sequences for Phosphoenolpyruvate carboxykinase**

>121879006

GCAGTGTGCATGTTACTCCACCTCCAAGAGTCAAGTGAAGCAGACTAGGAATGGTCATAGCTGAGTAACCATctaatcactatcaccaccaacaaacatCGTCAACATGGTGCTATCCACTAACAACTCTGCCAACAACATCATGAACCAGCCCAGACTGAGCAGTGTGCTGGCAGAGGCCTCCAGGGTCGCCTCTGAGGCAGCCAAGAAGAACCTCGACTTCCAGAGTCGCAGCCTCACTGTCATCCATGGCAAACTCTCCGACCTCAAGCCCAAGGTACGGACGTTCGTGGAGGAGAGCGCCAGACTGTGTCGACCCGAGAACGTACACATCTGTGATGGCAGCGAACGTGAACTGCGCCACCTGTTGAACGTGATGCAACAAGCGGGCATGATCGAGCATCTGCCCAAGTACAAGAACTGTTGGTTGGCTCGCACTGACCCCGGAGATGTTGCTCGCGTGGAAAGCAAAACGTTCATTGTGACCAAACACCGCAGAGACACCATCCCTACCCCTAAGGAAGGCGTCAAGGGCCTTCTGGGCAACTGGATGTCTTATGAGGATCTGAAAAGCGCCGTCCAGGAACGTTTCCCGGGCTGTATGTCTGGCAGGACCATGTATGTCGTGCCTTACTCCATGGGACCCGTAGGTTCTCCGCTCTCCAAGATCGGCGTTGAGGTCACAGACTCTCCCTATGTTGTGGCCTCTATGCGCACCATGACGCGGATGGGCGGTAAGGTGCTGGAAACTCTGGATCAAGAAGATTTCGTCAAGTGCCTTCACTCAGTTGGTTGTCCACTGCCCCTCAAGAAGACTCTTGTCAACAACTGGCCCTGTGACCCCGCACGAACCATCGTCACCCACGTCCCCGACACTAACGAGATCGTTTCCTTTGGATCAGGTTACGGAGGCAACTCGTTATTGGGCAAAAAGTGCTTTGCTCTGCGCATTGGCTCCAACATTGCCAGACGTGAAGGTTGGTTGGCTGAACACATGCTCATCCTGGGCATCACCAACCCTGAGGGCATCAAGAAGTACATTGCTGCAGCTTTCCCCTCTGCCTGCGGCAAGACCAACTTGGCTATGATGACACCTTCTCTGCCCGGCTACAAAGTAGAGTGCGTGGGTGATGACATCGCATGGATGAAATTTGACGAGGATGGCGTCTTGCGTGCCATCAACCCTGAGAATGGTTTCTTCGGCGTGGCTCCCGGCACCTCCATGCACACTAATCCTGTGGCCATGAAGACGGTTCTCTCCAACACAATCTTCACCAATGTTGCCAAGacgagtgatggaggtgtgttcTGGGAGGGCCTGGAGAAGGAGATTGACAACGACGTCACCATCACATCTTGGCTGGGAGATACAAATTGGAGTAAAGAATCTGGCAAGCCAGCCGCTCACCCTAACTCCAGATTCTGCACCCCAGCCGGCCAGTGTCCCATCATTGACCCCGCCTGGGAAGATCCTAAGGGCGTACCCATCTCTGCCATTCTCTTCGGTGGAAGACGTCCTCAAGGTGTGCCCTTGGTGTACGAGGCCTTCAACTGGAAGCACGGCGTCATGGTGGGAGGAAGCATGAGATCCGAGGCGACGGCAGCAGCTGAACACAAGGGTAAGGTCATCATGCACGACCCCTTCGCCATGAGACCATTCTTCGGTTACAACTTCGGCCACTACCTTCAACACTGGCTGAGTATGGAGACCCGCACAGACAAGCCTCTTCCCAAGATCTTCCACGTCAACTGGTTCCGCAAGAGCGAAAAGGGACGCTTCATCTGGCCAGGTTTCGGGGACAATGTGCGCGTCTTGGACTGGATCCTGAAGCGTGTTGACGGTGAGGACGTGGCAGAAGAGAGCGCCGTAGGACTCCTGCCAAAGGCGTCCTCCATCAACATGGATGGCCTGGAACACGAGAACGTTGATATGGATGAGCTGTTCAGCCTTCCTAAGGAGTTCTGGGAGCAGGAAGTGCGCGACATCGCTAAGTACTTTGACGAACAAGTTGGCGACGATGTGCCTAACGAGGTGCGGGAACAACTCAAGATGCTGGAGAAGAGAATCGAGAAGTCTTAGATGTGTGAGAGTTTTCATTGCAGGCAACAAGGAATGGTTACCTGTTATAGCGTACATAAGAAGCAATGACAATACTGATGGGGTATGATATCCCATCTCATATGATTGCATTTTTGATCACCGTTTCATTGTTTACATGTCATAAAAGTTCTCCAAATCCCCTCATCCACAAAATATCATATTATATCCCATACACTTTCATTTACTACTTCCAATTGTCATCTTCATATATTGGTTTTCATAGTTTTTATTTGTACATTTCACCACACTATGCATCTGAATGataatttctttgttttgtaatgCTGTGTATGTCAATGTTTTACGTGAATTGATGTTACATGCTTTTGCACAAGTGATCATGTGGTTACTCTATTGTAAAGCAAatacatattttatttttctatgaACCTAA

>121879005

tgtgtgtgttactccaccTCCAAGAGTCAAGTGAAGCAGACCAGGAATGGTCAACGTTAAGTAACCATctaatcactatcaccaccaacaaccatcgtCAACATGGTGTTTTCCACTAATAACTCTGCCAACAACAACATGAACCAGCCCAGACTGAGCAGTGTGCTGGCAGAGGCCTCCAGGGTCGCCTCTGAGGCAGCCAAGAAGAACCTCGACTTCCAGAGTCGCAGCCTCACTGTCATCCATGGCAAACTCTCCGACCTCAAGCCCAAGGTACGGACGTTCGTGGAGGAGAGCGCCAGACTGTGTCGACCCGAGAACGTACACATCTGTGATGGCAGCGAACGTGAACTGCGCCACCTGTTGAACGTGATGCAACAAGCGGGCATGATCGAGCATCTGCCCAAGTACAAGAACTGTTGGTTGGCTCGCACTGACCCCGGAGATGTTGCTCGCGTGGAAAGCAAAACGTTCATTGTGACCAAACACCGCAGAGACACCATCCCTACCCCTAAGGAAGGCGTCAAGGGCCTTCTGGGCAACTGGATGTCTTATGAGGATCTGAAAAGCGCCGTCCAGGAACGTTTCCCGGGCTGTATGTCTGGCAGGACCATGTATGTCGTGCCTTACTCCATGGGACCCGTAGGTTCTCCGCTCTCCAAGATCGGCGTTGAGGTCACAGACTCTCCCTATGTTGTGGCCTCTATGCGCACCATGACGCGGATGGGCGGTAAGGTGCTGGAAACTCTGGATCAAGAAGATTTCGTCAAGTGCCTTCACTCAGTTGGTTGTCCACTGCCCCTCAAGAAGACTCTTGTTAACAACTGGCCCTGTGACCCCGCCCGAACCATCGTCACCCACGTCCCCGACACTAACGAGATCGTTTCCTTTGGCTCGGGTTACGGAGGCAACTCGTTATTGGGCAAAAAGTGTTTTGCTCTGCGCATTGGCTCCAACATTGCCAGACGTGAAGGTTGGTTGGCTGAACACATGCTCATCCTGGGCATCACCAACCCTGAGGGCATCAAGAAGTACATTGCTGCAGCTTTCCCCTCTGCCTGTGGCAAGACCAACTTGGCTATGATGACACCTTCTCTGCCCGGCTACAAAGTAGAGTGCGTGGGTGATGACATCGCATGGATGAAATTTGACGAGGATGGCGTCTTGCGTGCCATCAACCCTGAGAACGGGTTCTTCGGCGTGGCTCCCGGCACCTCCATGCACACTAATCCTGTGGCCATGAAGACGGTTCTCTCCAACACAATCTTCACCAATGTTGCCAAGacgagtgatggaggtgtgttcTGGGAGGGCCTGGAGAAGGAGATTGACAACGACGTCACCATCACATCTTGGCTGGGAGATACAAATTGGAGTAAAGAATCTGGCAAGCCAGCCGCTCACCCTAACTCCAGATTCTGCACCCCAGCCGGCCAGTGTCCCATCATTGACCCCGCCTGGGAAGATCCTAAGGGCGTACCCATCTCTGCCATTCTCTTCGGTGGAAGACGTCCTCAAGGTGTGCCCTTGGTGTACGAGGCCTTCAACTGGAAGCACGGCGTCATGGTGGGAGGAAGCATGAGATCCGAGGCGACGGCAGCAGCTGAACACAAGGGTAAGGTCATCATGCACGACCCCTTCGCCATGAGACCATTCTTCGGTTACAACTTCGGTCACTACCTTCAACACTGGCTGAGTATGGAGACCCGCACAGACAAGCCTCTTCCCAAGATCTTCCACGTCAACTGGTTCCGCAAGAGCGAAAAGGGACGCTTCATCTGGCCAGGTTTCGGGGACAATGTGCGCGTCTTGGACTGGATCCTGAAGCGTGTTGACGGTGAGGACGTGGCAGAAGAGAGCGCCGTAGGACTCCTGCCAAAGGCGTCCTCCATCAACATGGATGGCCTGGAGCACGAGAACGTTGATATGGATGAGCTGTTCAGCCTTCCTAAGGAGTTCTGGGAACAGGAAGTGCGCGACATCGCAAAGTACTTTGACGAACAAGTTGGCGACGAACTGCCTAACGAGGTGCGGGAACAACTCAAGATGTTGGGGAAGAGAATCGAGAAGTCTTAAAGTTTAATACATAAAGGCTCTTTGCCGCGGTAACACAAAGCTGTGAGCTGTTGTGTGTAATATCTGAAGCTATGACAATACAGATGGGGTGTGGTATCCTATCTCTGGTGGAAACATTCATTATCATTTAACTCAAGACAATATTGTTTTCATAAAACTCATCATCAAGAATCTATAACTCCTAAAAGAAAATTATTATCTATCACTGTCAGTTTCATCAAGATATTCATTCATACAATTCTTCACTACACATTCTTCACATTATCATCTGTCAATGAACAAATGTAATTGGTTATTGTAATGCTGTGCAGTACAATATTTTACGTGGAGTTTACCGCGTTGTTTTTAGATACGTTATCGTGTTATTActttatattataataaatatatattttttctatgaa
