# Supplementary material for: Rearing condition influences gene expression in postlarval American lobster (Homarus americanus)
Source: PLoS One. 2024 Jul 18;19(7):e0307169. doi: 10.1371/journal.pone.0307169 (PMC11257295; doi:10.1371/journal.pone.0307169)
Supplement: Attachment — Submitted filename: Response to Reviewers copy.docx [file pone.0307169.s006.docx]

We thank the reviewers for the comments and suggestions and outline below how we addressed them:

6. Review Comments to the Author

Reviewer #3: General feedback:
This is a review of the revised manuscript, “Rearing condition influences gene expression in postlarval American lobster (Homarus americanus),” submitted by Jane et al. to PLOS ONE. This is an interesting paper that explores the differential gene expression of lab-reared vs. wild-caught postlarval lobster in the context of diet and thermal stress. This is a well-written and focused paper that contributes to our broader understanding of lobster biology while providing a strong evidence for scientists to consider how diet and rearing conditions (i.e., lab-reared vs. wild-caught postlarvae) impact experimental results. The authors do an excellent job addressing the insightful comments/feedback from the previous reviewers, which I strongly believe helped to improve the overall message and conclusions of the manuscript. The authors managed to maintain their point of view while ensuring the results were clearly explained through the inclusion of additional and/or modified figures and tables.

*Thank you for this positive assessment of our revised manuscript. The reviewers’ comments were most helpful in strengthening it.*

I do have a few minor suggestions (see below), but overall think this was an excellent revision that addressed the reviewers’ feedback while strengthening the central conclusions – well done!

Specific feedback:
Abstract:
• Lines 28-30: I agree that this should be included in the abstract, but find it a bit hard to read. Perhaps specify “wild-caught”? It also seems odd to start a sentence with a number (line 29)?

*We adjusted the wording to make it easier readable*

• Line 33: Since the abstract is what draws readers in, perhaps you could include a description of the FoxO signaling pathway.

*We included a very brief description of the FoxO pathway in the abstract*

Introduction:
Line 59: The way this sentence is written suggests that development time increases (i.e., is longer) with warming, but you clearly state that development time is reduced (i.e., faster) under warming at the end of the paragraph – perhaps re-word to clarify your point?

*The word “decreased” was missing for the development time. Thanks for catching this*

Methods:
• Line 97: Should Waller et al. (2023) be a numerical citation?

*We removed this citation as it is not necessary*

• Lines 111-112: It would be helpful to include the average sizes rather than just reporting the percent increase in size of postlarvae fed zooplankton vs. brine shrimp.

*We now include the average weight of both groups of larvae*

• Line 119: I think there is a typo (misplaced period).

*fixed*

• Line 120: Is ambient referring to temperature at the collection site?

*Temperature of the flow-through tank fed by the Damariscotta River estuary; we clarify this now in the manuscript*

• Lines 121-122: As stated above, it would be helpful to include average sizes of postlarvae in different treatments rather than just the percent increase in size of wild-caught individuals.

*We now include the average weight of both groups of larvae*

• Line 126: I think you took the explanation out, but why did you only expose zooplankton-fed postlarvae to cold stress?

*This was done simply for logistical reasons (limited time and funding for additional experiments). At the time when we made the decision, it seemed a good choice. After analyzing the data, we certainly wished that we had done these experiments as well, but now it is unfortunately too late. We refrained from explaining this (difficult) choice in the manuscript.*

• Lines 129-130: You might consider adding a sentence explaining how different the temperature treatments were from natural conditions. I’d also explain what “immediate” means - did you just change the heater to 26°C from 18 (or lower), or was there an increase/decrease over a day? I imagine it could be really stressful to go through immediate shifts, and that this might influence your results?

*We added the respective details*

• Line 139: Should this be a numerical citation?

*Fixed*

Results:
• Line 182: I would spell out the number to start the sentence.

*We reworded the sentence so that it does not start with a number*

• Line 213: I’m not sure I understand why you called out heat shock protein 90 here when it’s not mentioned in the discussion?

*We removed the mentioning of HSP70 here*

• Lines 234, 255, and 266: It would be helpful to clarify that this was at ambient temperature in the subheadings.

*Adding this information into the subheadings would make them very long. Therefore, we added the description of this being larvae reared at ambient temperature into the first sentences of the respective paragraphs, following the subheadings.*

Discussion:
• Line 383: This seems incomplete – maybe change to “wild-caught” or “wild postlarvae”.

*Fixed*

• Line 414: Should “Zhang et al. 2023” be a numerical citation?

*Fixed*

• Line 446: I think there may be a typo (misplaced underscore between “data of”).

Fixed
